# Analysis of issues related to nursing law: Examination of news articles using topic modeling

**DOI:** 10.1371/journal.pone.0308065

**Published:** 2024-08-22

**Authors:** JooHyun Lee, Hyoung Eun Chang, Jaehyuk Cho, Seohyun Yoo, Joonseo Hyeon

**Affiliations:** 1 Graduate School of Public Policy & Civic Engagement, Kyung Hee University, Seoul, Republic of Korea; 2 College of Nursing, Research Institute of Nursing Science, Jeonbuk National University, Jeonju, Jeollabuk-do, Republic of Korea; 3 Department of Software Engineering & Division of Electronics and Information Engineering, Jeonbuk National University, Jeonju, Jeollabuk-do, Republic of Korea; 4 Department of Software Engineering, Jeonbuk National University, Jeonju, Jeollabuk-do, Republic of Korea; University of Foggia: Universita degli Studi di Foggia, ITALY

## Abstract

**Purpose:**

The objective of this study was to analyze proposed Korean nursing legislation as depicted in newspaper articles, to highlight issues related to the legislative process for this potential law, and to better understand social awareness regarding this matter.

**Methods:**

The study focused on articles from 11 leading newspapers in Korea, published between February 2020 and August 2023, that pertained to nursing legislation. The articles were retrieved from the BigKinds database. Following text preprocessing, analytical methods including term frequency-inverse document frequency were employed, along with latent Dirichlet allocation (LDA), for word and topic modeling analysis. Additionally, LDA was applied across time periods to examine temporal changes in topics.

**Results:**

Following preprocessing, a total of 7,967 words were extracted from the 991 articles selected for analysis. The primary themes identified in newspaper articles concerning the nursing legislation were organized into three main topics: 1) the necessity and impact of enactment of the nursing law, 2) the political context surrounding enactment of the law, and 3) the conflicts between and actions of healthcare organizations related to enactment of the law.

**Conclusions:**

The findings confirmed that media coverage regarding the proposed nursing legislation primarily concentrated on the political and social conflicts associated with the law’s passage, rather than its necessity and substance. More compelling evidence must be presented concerning the influence of the nursing workforce and the work environment of nurses on patient safety and health outcomes. Additionally, strategies should be devised to improve public comprehension of the nursing law’s provisions.

## Introduction

Amidst the accelerating changes in population structure due to low birth rates and aging, a corresponding shift has occurred in disease patterns and the utilization of medical care across the globe [1]. These trends are driving an increased demand for nursing professionals. To prevent the deterioration of health insurance finances, which are strained by rising medical costs for the elderly, a transformation of the healthcare system is necessary. Specifically, the focus should shift from hospital-based disease treatment to community-centered disease prevention, management, and health promotion. Concurrently, the scope of nursing practice is expanding to include schools, nursing homes, facilities for the elderly and disabled, and industrial settings, necessitating a broader range of roles for nurses [2]. However, in South Korea, the medical law enacted in 1962 has retained a scope of application limited to medical institutions, even after major revisions in 1973 and 2007. Specifically, nurses’ responsibilities have been restricted to “assistance in medical treatment,” and even a 2015 amendment failed to encompass the full spectrum of nursing activities [3]. Thus, regulations pertaining to nursing personnel in settings outside of medical institutions, such as communities, are dispersed across more than 10 different laws. These include the Regional Health Act, the School Health Act, the Long-Term Care Insurance for the Elderly Act, and the Special Act on Rural Health and Medical Services [4]. This fragmentation fosters confusion among nurses when carrying out their duties and hinders the efficient management of the nursing workforce.

Discussions in Korea regarding the creation of a dedicated nursing law began in earnest during the 1970s. However, the origins of legal regulations pertaining to nursing can be traced to the enactment of the Joseon Midwifery Rules and the Joseon Nursing Department Rules in 1914 [5]. These laws initially established an independent licensing system for the profession. However, with Japan’s introduction of the Joseon Medical Ordinance in 1944, the legal framework for nursing was integrated with that of physicians, dentists, and practitioners of oriental medicine. The subsequent National Medical Service Act laid the foundation for the medical laws that regulate all medical practices to this day. Japan enacted separate laws for public health nurses, midwives, and nurses in 1948, responding to specific healthcare needs at the time. Similarly, the United States began regulating nurses’ scope of work and licensing on a state-by-state basis in the 1940s [6]. Over 90 countries worldwide, including Organization for Economic Co-operation and Development (OECD) members, regulate nurses’ licensure, qualifications, and scope of work through nursing laws. These countries are actively working to enhance the advanced practice nurse system, improve the work environment for nurses, and increase nurse staffing levels [7–9]. These efforts have led to discussions and the implementation of practical policies aimed at advancing nursing practices at a more specific level. The objective of the nursing law is to enhance patient safety and public health by ensuring the provision of high-quality nursing services both within and beyond medical institutions [10], thereby safeguarding the rights and interests of citizens as consumers of healthcare services.

Meanwhile, despite the annual graduation of more than 20,000 nurses from training, the ratio of nurses actively engaged in medical settings per 1,000 people in Korea remains only approximately half of the average among OECD countries [11]. This discrepancy has been attributed to issues such as shift work, work-family conflict, role conflict among nurses, excessive workloads, and workplace bullying, all of which contribute to difficulties in nurse retention and compromise patient safety [12]. Consequently, calls have been increasing for active government intervention to address these concerns. Nurses have advocated the establishment of new nursing legislation to systematize present nursing-related law, which is considered out of touch with the realities of the profession. They argue that such a law is necessary to define the scope of nursing practice by acknowledging the specificity and uniqueness of the work, and to enhance the training, supply, demand, and working conditions of the nursing workforce.

The Korean Nurses Association has been continually engaged in initiatives for this purpose, with actions such as adopting a resolution to call for the creation of a nursing law, organizing policy forums, reviewing foreign nursing laws for comparative legal analysis, and submitting legislative recommendations to the government [13]. The emergence and spread of new infectious diseases, including severe acute respiratory syndrome (SARS) in 2003 and Middle East respiratory syndrome (MERS) in 2015, have highlighted the critical shortage of healthcare workers, particularly in clinical nursing. This shortage has been acknowledged as a societal issue. Consequently, the introduction of a nursing law was suggested as an urgent policy measure to improve the public healthcare system and ensure the availability of qualified nurses. However, the proposed bills failed to reach the relevant standing committees for consideration and were ultimately discarded at the end of the legislative session. Amidst these developments, the global coronavirus disease 2019 (COVID-19) pandemic has heightened public interest in, and awareness of, the nursing workforce and working conditions. A study that analyzed the frequency of nursing-related topics on internet forums and social media platforms found that discussions about nurses displayed a 1.38-fold increase following the outbreak of COVID-19 [14]. This surge in public interest served as a catalyst, and in 2021, nursing legislation in Korea was proposed for the third time. The bill was directly referred to the plenary session of the relevant standing committee and, with the support of the opposition party, was placed on the agenda and passed. Despite this progress, the ruling party and other medical groups that were against the enactment of the nursing law intensified their opposition. Ultimately, the bill was defeated when the Korean president exercised veto power.

A policy is a strategy implemented by a government agency, characterized by its objectives, methods, and aims. Before a policy is established, societal challenges emerge as social issues, ascend to the public sphere, and subsequently evolve into policy agendas [15]. In this context, the media plays a crucial role in elevating a social problem to the public agenda by fostering consensus, not only among stakeholders but also within the public, that the government must address the issue. The reason for this is that the media not only provides factual information, but also influences users’ attitudes toward social and political issues through its reporting and editorial decisions [16]. In other words, in the process of setting the agenda, the media serves as a channel for mediating between representative or political institutions and the public’s interests, or it creates and spreads its own agenda [14]. This dynamic arises because the media reflects public opinion while also having the power to shape or steer it [17].

In light of the global COVID-19 pandemic, there has been a renewed examination of the influence of media coverage on public opinion. Techniques such as text mining and topic modeling have been employed to analyze extensive volumes of unstructured data sourced from press articles and social media platforms [18]. These unsupervised machine learning methods have the ability to mitigate researcher subjectivity inherent in traditional thematic analysis, thereby achieving objective outcomes in macroscopic analyses [19]. Unsupervised learning derives probabilistic clusters based on the data itself, enabling exploratory analyses in social science research. This method has been widely used to make descriptive sense of unstructured big data, such as identifying emerging policies related to health, climate, and other areas, assessing government policy positions, and analyzing trends in media coverage and public opinion shaping the agenda [20–22]. In the realm of nursing, numerous studies have analyzed the portrayal of nurses in media coverage and public discourse, aiming to understand the prevailing perceptions of nurses among the general populace [13, 23].

Therefore, by looking back on the attempt to enact this nursing law in 2021, which was the only nursing law proposed in the previous National Assembly term in Korea to pass the plenary session, it is possible to determine how public opinion formed and evolved, as well as identify the primary concerns, by analyzing the content, keywords, and related topics regarding media reports about the nursing law’s enactment. Thus, the research questions of this study were “What issues were discussed in newspaper articles related to the legislative process of the Korean nursing law?” and “What were the reactions of the media and society regarding the enactment of the Korean nursing law?” Accordingly, the purpose of this study was (1) to use newspaper articles to examine the portrayal of the proposed nursing law, (2) to highlight related issues within the legislative process, and (3) to better understand future directions in the legislative process.

## Methods

### Study design

This exploratory study employed text mining, keyword analysis, and topic modeling based on machine learning to analyze the principal keywords, structure, trends, and topics present in newspaper articles pertaining to nursing law.

### Data collection

The study focused on articles from 11 leading newspapers, published between February 2020, when the domestic spread of COVID-19 began in earnest, and August 2023, when the rejection of the Nursing Act precipitated a nurses’ strike. A search was conducted on Big Kinds, a news-centric open big data platform operated by the Korea Press Foundation. Big Kinds is a database that collects articles from 104 media organizations published in Korea and provides archiving, searching, and other services using the collected big data. The 11 leading newspapers were selected considering their media impact and influence on public opinion, as they were the only national dailies. The search terms used were “nursing law” AND (“nurse” OR “nursing workforce” OR “nursing association”). To finalize the search criteria, the contents of each retrieved article were randomly scrutinized using terminology related to nursing legislation. When a search was conducted solely for "nursing law," it was observed that the content of the articles often did not pertain directly to nursing law, with a high proportion merely mentioning the term. Consequently, it was determined that the search terms should encompass terms such as "nurse," "nursing personnel," or "nursing association," which yielded results more directly relevant to nursing law. For a total of 1,078 search results from October 10 to 11, 2023, we crawled the articles by their URLs using Python web scraping. We then retrieved the corresponding articles from each media outlet and verified the retrieved content against the article URLs found in Big Kinds, making adjustments as necessary. Articles were excluded from the study if they were 1) photographic or breaking news pieces consisting solely of titles and photos with no text, 2) identical reports published multiple times by the same media outlet, or 3) articles not pertinent to the nursing legislation. Two researchers independently reviewed the content of the excluded articles. Instances of the same leading news article published in multiple newspapers were not treated as duplicates, as each newspaper’s judgment of the article as a leading one was significant. Furthermore, the reproduction and spread of articles are themselves important factors in determining media coverage trends. Through this selection process, a dataset of 991 articles was finalized for analysis. Before the study began, an exemption from review was granted by the institutional review board (IRB) of the affiliated institution (IRB No. 2023–167).

### Data analysis

#### Text preprocessing

The analysis included 991 articles that underwent morpheme extraction using the Mecab tool from the KoNLpY library, which is a Korean-language information processing package for Python. Morpheme extraction is the process of classifying words into so-called minimum meaningful units, based on both lexical and grammatical meanings. Words were then extracted by assigning parts of speech to the morphemes by the Mecab morpheme extractor. Among the separated words, nouns with identical meanings but different spellings were consolidated into representative terms using synonyms. For instance, variations like “nursing association” or “nurse assoc.” were standardized to “Korean Nurses Association,” while “clinical support nurse” or “physician assistant” were both categorized under “PA.” Conversely, words lacking meaningful content were classified as stopwords and subsequently removed. Common words in newspaper articles that carry no relevant meaning, such as “front,” “afternoon,” “moment,” and “point,” were eliminated. Reporters’ names and email addresses were similarly treated as stopwords and removed. In creating the dictionary for similar and excluded words, those occurring the most often were prioritized using frequency analysis. Two nursing and software engineering researchers repeatedly reviewed and verified the preprocessing of these data, reaching a final consensus on the results. Frequency analysis and topic modeling were conducted using the algorithm offered by Scikit-learn, a Python-based machine learning library.

#### Topic modeling analysis

Three distinct modeling approaches were utilized for word and topic analysis. The first method, the most straightforward form of topic modeling used, combined term frequency-inverse document frequency (TF-IDF). In this approach, TF is the number of times a specific word appears in a specific document, and IDF is the reciprocal of the number of documents in which a specific word appears. The importance of each word is quantified through the creation of a document-term matrix, in which the frequency of a particular word is weighted by the inverse of the number of documents in which that word appears. A higher TF-IDF value indicates that a word is more important in the documents [24].

The second method applied was latent Dirichlet allocation (LDA), a model that assigns latent topics distributed according to a Dirichlet distribution. It is based on the concept that each document contains words from multiple topics, where the proportion of each topic in each document is different, but the topics are the same across all documents [25]. LDA employs a probabilistic approach based on unsupervised learning. It operates under the assumption that documents are composed of randomly mixed topics, each represented by a distribution of words. LDA has been recognized as particularly effective for topic modeling in collections of documents that contain a diverse vocabulary [26]. In this context, the Dirichlet distribution, denoted by θ and parameterized by α, gives rise to topics that follow a multinomial distribution with θ as the parameter. Words then manifest as conditional probabilities given the topic and a second parameter, β [26]. The values of the hyperparameters α and β were determined using a grid search strategy. Here, α was tested at the number of topics (K), 50 divided by K, and 5 divided by K. Meanwhile, β was set at 0.1, in line with prior research [27]. The variables are defined in Table 1.

**Table 1:** LDA variables and parameters.

Variable	Description
K	Represents the number of topics
M	Represents the number of documents
α	Represents a previously defined weight for a Dirichlet distribution relative to a topic K
β	Represents a previously defined weight for a Dirichlet distribution relative to word in a topic
θ	Represents the distribution of topics in documents

Coherence and perplexity were used as evaluation indices for hyperparameter optimization. Coherence quantifies the semantic similarity among words that exhibit high values within a single topic, with a higher coherence score indicating greater semantic consistency [28]. Perplexity measures the predictive accuracy of a probability model against observed data; a lower value indicates that the topic model better reflects the document [26]. The algorithm-based measurements of each metric all converged monotonically, and the hyperparameters were optimized to the point in time immediately after the largest rate of change. We used tomotopy for LDA modeling and training, genism for hyperparameter optimization, and pyLDAvis for visualization.

The words within each resultant cluster were validated by two nursing researchers and interpreted in connection with the respective topic. Subsequently, upon revisiting the source article for each word, the final theme for each cluster was determined through mutual agreement. Through iterative review and discussion of the topic modeling outcomes, efforts were made to minimize researcher subjectivity, ensuring that the named topics effectively encapsulated the words within each cluster in a cohesive and lucid manner.

The third method assumed that the entire dataset constituted a single topic and performed LDA for each period to check for changes in the proportion of keywords in the articles. The timeline was segmented into three periods, aligned with pivotal moments in the legislative journey of the nursing legislation: 1) the span from February 1, 2020 to April 26, 2023, prior to the bill’s passage in the plenary session (period 1), 2) the interval from April 27, 2023, after the passage of the bill, until May 15, 2023, leading up to the president’s veto (period 2), and 3) the stretch from May 16, 2023, after the veto to August 31, 2023 (period 3). The counts of articles examined for each period were 387, 306, and 298, respectively.

## Results

### TF-IDF

Among the 991 articles that were analyzed, variations were observed in the number of articles pertaining to nursing law across different media outlets. Notably, the top three media outlets accounted for approximately 47% of all nursing law-related articles. The majority of articles comprised news pieces, which objectively presented factual information, or explanatory articles clarifying aspects related to the enactment of the Nursing Act. Approximately 10% of the articles were opinion pieces, including editorials, columns, and contributions. After preprocessing the 991 articles selected for analysis, a total of 7,967 words were ultimately extracted. Frequency analysis revealed that the term “nursing law” appeared 7,213 times, “nurse” 5,716 times, “medicine” 4,438 times, “president” 3,221 times, and “physician” 2,633 times (Table 2). To assess the importance of these words, TF-IDF was employed for the document-word matrix.

**Table 2:** Term frequency and term frequency-inverse document frequency of 15 keywords.

Rank	Term	TF	Term	TF-IDF
**1**	nursing law	7,213	nursing law	109.4
**2**	nurse	5,716	nurse	85.2
**3**	medicine	4,438	medicine	73.3
**4**	president	3,221	president	68.3
**5**	physician	2,633	veto	53.9
**6**	congress	2,537	physician	47.5
**7**	enactment	2,332	Korean Nursing Association	47.1
**8**	veto	2,273	congress	46.5
**9**	nursing	2,144	Democratic Party	44.3
**10**	Korean Nursing Association	2,087	enactment	43.7
**11**	people	1,910	exercise	43.2
**12**	Democratic Party	1,893	nursing	43.0
**13**	exercise	1,834	plenary session	38.9
**14**	work	1,774	people	37.4
**15**	plenary session	1,661	bill	37.2

### LDA

**Fig 1:**
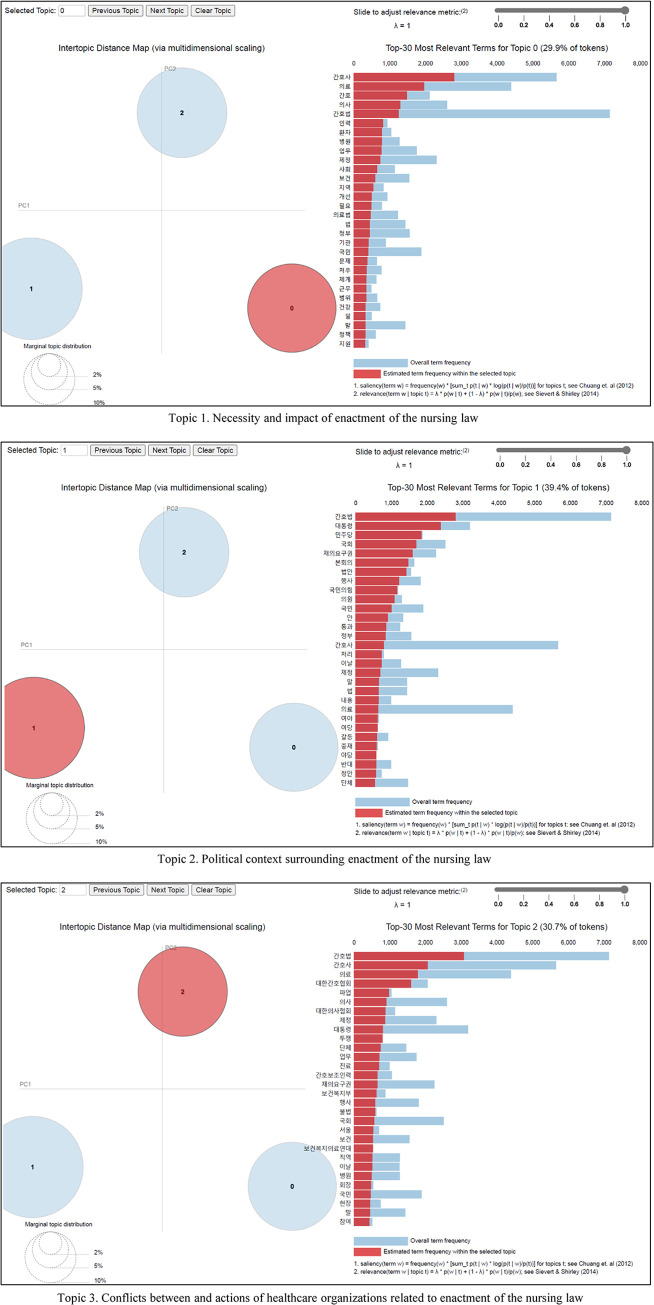
Results of latent Dirichlet allocation topic modeling.

Upon validating the LDA learning model through coherence and perplexity measures, the hyperparameter α was established at 5.56, and the number of topics was determined to be 3. The outcomes of the analysis were graphically represented with the aid of the LDAvis package for R (R Foundation for Statistical Computing, Vienna, Austria) (Fig 1).

The first topic, comprising 29.9% of the total data, included keywords associated with various components of the healthcare system influenced by the introduction of the nursing law. These keywords encompassed terms such as “nurse,” “medicine,” “physician,” “workforce,” “patients,” “hospital,” and “work.” Consequently, this topic was termed “the necessity and impact of enactment of the nursing law.” The second topic represented 39.4% of the total and featured keywords pertaining to legislative and political processes, including “president,” “Democratic Party,” “congress,” “veto,” and “plenary session.” This topic was thus labeled “the political context surrounding enactment of the nursing law.” Keywords for the third topic, which constituted 30.7% of the total, were related to the discord between and actions taken by the nursing and medical communities during the legislative process. These included “nurse,” “Korean Nurses Association,” “strike,” “physician,” “Korean Medical Association,” and “protest.” The topic was accordingly named “conflicts between and actions of healthcare organizations related to enactment of the nursing law” (Table 3).

**Table 3:** Major topics and keywords identified through latent Dirichlet allocation topic modeling.

Topics	Token (%)	
Necessity and impact of enactment of the nursing law	29.9	nurse, medicine, nursing, physician, workforce, patients, hospital, work, society, healthcare
Political context surrounding enactment of the nursing law	39.4	president, Democratic Party, congress, veto, plenary session, bill, exercise, People Power Party, member of congress, pass
Conflicts between and actions of healthcare organizations related to enactment of the nursing law	30.7	nurse, Korea Nursing Association, strike, physician, Korean Medical Association, enactment, protest, organization, medical treatment, nursing assist workforce

### LDA by periods

The analysis was segmented into three periods to assess shifts in the proportions of keywords defining the subject matter, in relation to pivotal moments in the relevant legislative process. Articles that appeared in April 2023, coinciding with the passage of the nursing bill during the full assembly of Congress, and in May 2023, when the President issued a veto, comprised 71% of the overall total.

**Fig 2:**
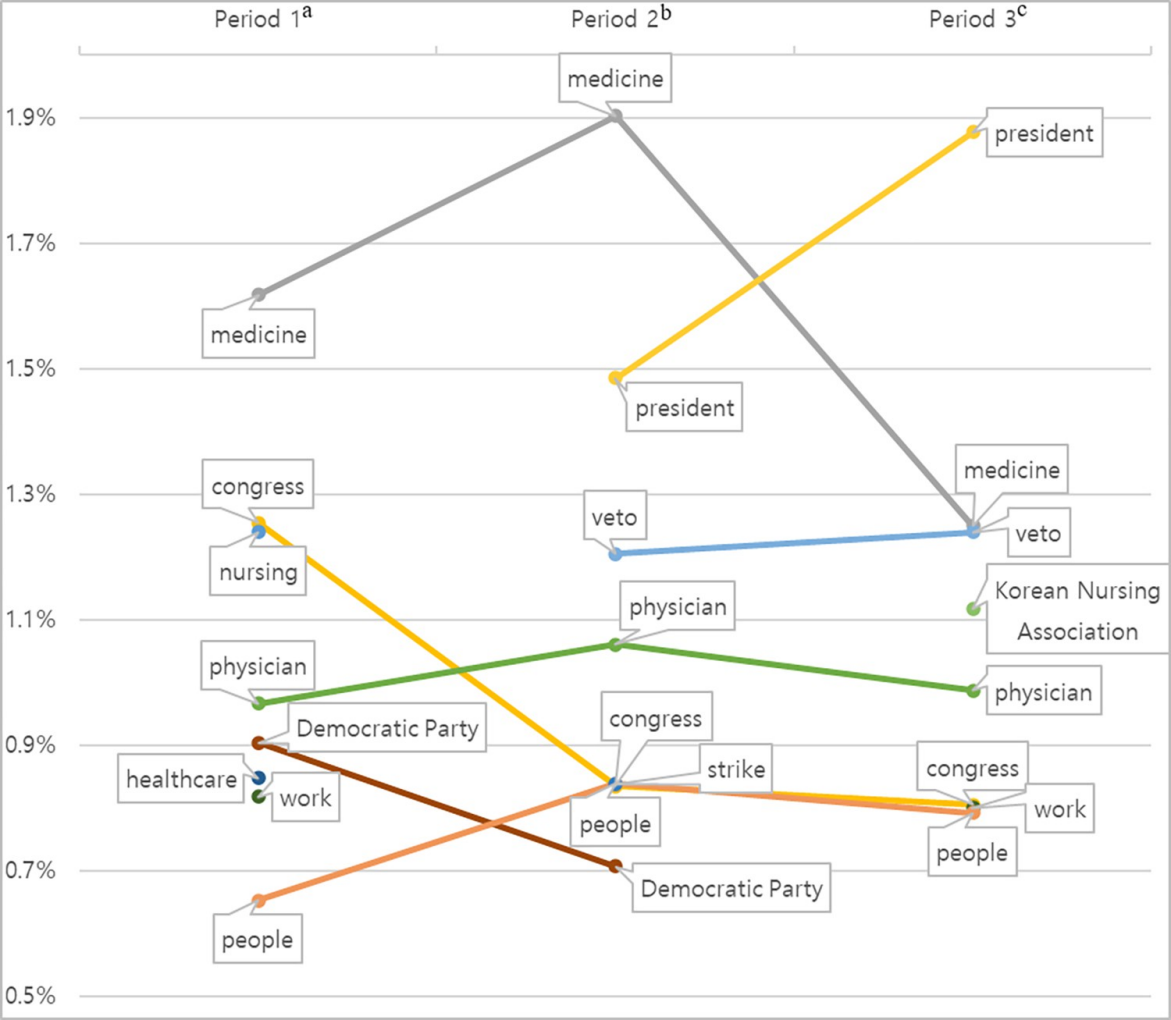
Proportions of keywords across three periods, as determined by latent Dirichlet allocation. ^a^February 1, 2020 to April 26, 2023, prior to the nursing law’s passage in the plenary session. ^b^April 27, 2023 to May 15, 2023, leading up to the president’s veto. ^c^May 16, 2023 to August 31, 2023.

Within all periods, the terms “nursing law” and “nurse” displayed the largest proportions with unchanged rankings; therefore, they were omitted from the analysis. The next most prevalent words were graphically represented, as shown in Fig 2. The terms “medicine,” “physician,” “congress,” and “people” were confirmed among the top words in all periods. The term “medicine” reached its peak in period 2, accounting for 1.9% of the data, while “physician” saw a gradual increase, peaking at 1.1% in period 3. Conversely, both “congress” and “people” displayed their lowest proportions in period 3, at 0.8% each (0.8% each). Words such as “nursing,” “healthcare,” and “work” appeared in period 1 (at respective proportions of 1.2%, 0.8%, and 0.8%), then disappeared in period 2. Similarly, “Democratic Party” appeared in period 1 with a proportion of 0.9%, declined to 0.7% in period 2, and was absent in period 3. In contrast, period 2 saw the emergence of new terms like “president,” “veto,” and “strike,” with proportions of 1.5%, 1.2%, and 0.8%, respectively. The term “president” showed a particularly sharp increase in period 3, reaching 1.9% and ranking as the third most prevalent term after “nursing law” and “nurse.”

## Discussion

In this study, the primary topics of newspaper articles concerning the proposed nursing legislation were categorized into three areas: the necessity and impact of enactment of the nursing law, the political context surrounding enactment, and the conflicts between and actions of healthcare organizations regarding enactment. Of these, the topic that occupied the largest share of coverage was the political context surrounding the law’s enactment, while the topic that received the least attention was the necessity and impact of enactment of the nursing law. This indicates that media coverage was more focused on the disputes between political parties and between the legislature and the government than on the underlying reasons for the proposed law’s introduction or its potential effects on the public and the healthcare system. This emphasis is further evidenced by the finding that terms such as “president,” “congress,” “veto,” “Democratic Party,” “plenary session,” and “government” ranked among the top 15 most frequently mentioned words in the study findings and the finding that according to TF-IDF, the importance of words such as “nursing,” “people,” and “work” decreased further.

The fact that terms related to the political context comprised the largest proportion of coverage is linked to the prominence of issues surrounding conflicts between healthcare organizations, which ranked immediately behind. This subject area predominantly included terms such as “Korean Nurses Association,” “strike,” “physician,” “Korean Medical Association,” “protest,” “organization,” and “nurse assistant workforce.” This indicates that the primary focus was on the disputes between healthcare and medical organizations that arose following the introduction of the nursing legislation. Moreover, this implies that the nursing community’s actions and platform in support of the nursing law, including issues such as illegal medical practices by nurses, compliance protests, and strikes for annual leave, were less strongly emphasized than conflicts with the medical community. This is further evidenced by the frequency of word usage, with “medicine” and “physician” appearing more often than “nursing” or “healthcare.” Additionally, in the TF-IDF analysis, they were also ranked higher than “nursing” or “Korean Nurses Association.” Ultimately, we determined that most articles concerning the nursing law’s enactment focused on the confrontation between political parties and healthcare/ medical organizations over the advantages and disadvantages of the law’s introduction, with the core issues addressed by the law receiving a comparatively small share of attention. This trend spurs questions about whether media reports have effectively fulfilled their role in providing the public with accurate information regarding the necessity of the nursing law, its fundamental objectives, and realistic associated concerns.

This pattern was further examined by analyzing the temporal shifts in key terminology. With the exception of “Nursing Law” and “Nurse,” the proportions and rankings of which remained fundamentally unchanged across study periods, terms such as “medicine,” “congress,” “nursing,” “physician,” “Democratic Party,” “healthcare,” “work,” and “people” were the top words in the first period. However, a significant shift was observed in the dominant words observed during the second period. In a span of fewer than 20 days, across over 300 articles, terms that pertained to the necessity or specifics of the nursing law’s enactment, such as “nursing,” “healthcare,” and “work,” vanished from the rankings. Instead, words like “veto,” “strike,” and “president” emerged and ascended to the top of the list. Throughout all periods, words such as “physician,” “medicine,” and “congress” were consistently present, whereas “Korean Nursing Association”—a term emblematic of the nurses’ stance—only surfaced in the third period. In essence, at the peak of media and public attention regarding the proposed nursing law, the primary words and themes in media reports centered on the political and societal disputes related to the law’s passage.

In democratic nations, the passage of new legislation is achieved through legislative and administrative processes. Various interest groups, potentially impacted by the new law, exert influence to further their aims. It is crucial for legislative and administrative bodies, including members of Congress and the President (who wield direct authority), to actively engage in this discourse. However, media coverage that focused predominantly on the political and social disputes surrounding the enactment of nursing legislation is believed to have significantly influenced the public’s understanding in the present case. This coverage likely failed to effectively communicate the fundamental reasons and necessity for the nursing law, increased public fatigue with the subject, and ultimately may have caused individuals to become disengaged from this critical issue.

Additionally, in media coverage pertaining to the passage of the nursing law, terms associated with physicians and the medical community were ranked highly in frequency, importance, and proportion. Regarding the frequency of appearance, “medicine” appeared more often than “nursing,” and neither the nurse assistant workforce nor nursing assistant associations appeared within the rankings. Furthermore, the results of the TF-IDF analysis indicated that “medicine” and “physician” were deemed more important than “nursing” and “people.” Despite the enactment of the nursing law primarily impacting nurses and recipients of nursing in communities who fall outside the jurisdiction of the medical law, the discourse in the articles was largely dominated by the perspectives of the medical community.

To interpret these results, it is essential to consider both the historical context and the theoretical framework in accordance with policy trends. The concept of the modern nurse emerged from the differentiation of roles between physicians and nurses. As patients began to seek care in hospitals, a need arose for comprehensive patient care, which led to the delineation of responsibilities: physicians became focused on diagnosing and treating diseases, while nurses adopted the roles of supporting physicians and providing holistic care to patients [8]. Nurses sought to specialize within their field as it emerged from this divergence of roles. However, this specialization sometimes led to conflicts with physicians, who viewed nurses primarily as their assistants [8]. For instance, the medical community raised concerns that the 2022 revisions to the legal scope of practice for advanced practice nurses, which allowed them to operate under a physician’s guidance or prescription, could compromise the safety of the physician’s practice. Furthermore, some argued that the proposed nursing legislation, which aimed to permit all individuals to receive nursing services at medical institutions “and [in] the community,” infringed on the public’s right to health by enabling nurses to establish independent practices, without physician oversight [29]. These arguments are rooted in the historical and perceptual background previously described.

While issues that serve the interests of established groups tend to easily gain a place on the agenda, matters that would alter existing privileges and benefits struggle to do so, despite their social importance. This phenomenon is known as non-decision making [30]. The preferences and intentions of political elites are crucial in these instances of non-decision making, yet the social structure also plays a considerable role in determining whether a topic will garner public attention [31]. The unique challenges faced by nurses, such as the work environment, conditions within medical institutions, and staffing concerns, have started to draw public interest due to the unusual circumstances of the COVID-19 pandemic [32]. However, we posit that the details and context surrounding the proposed nursing legislation were so complex that it failed to sustain public engagement. In this context, the medical community, which is a powerful interest group, had its concerns disproportionately represented. Meanwhile, the issues pertinent to the nursing community, which is at the heart of the nursing law, are perceived to have failed to gain a prominent foothold within the public discourse.

The government, ruling party, opposition party, and civil society held divergent views on the enactment of the nursing law, rendering politicization of the issue unavoidable. Nevertheless, this study has established that the keywords found in newspaper articles reflect the political and social conflicts associated with the proposed nursing legislation, while the fundamental aspects of the law were downplayed and received minimal attention in the coverage. These findings have multiple implications concerning the legislative process for the proposed nursing law.

First, it is necessary to evaluate whether media coverage has adequately addressed the specific provisions of the nursing law and the need for its enactment. Media outlets should strive to provide the public with accurate information about the nature of the nursing law and its necessity, rather than merely echoing the demands of interest groups involved in its creation. By presenting factual content, the media can establish a forum for debate and help shape public opinion, a critical step toward achieving societal consensus. Second, it is necessary to assess whether the perspectives of the nursing community—which is directly affected by the nursing law—have been sufficiently represented and prioritized. Moving forward, the nursing community should devise a strategy that ensures their viewpoints are reported in the media with greater objectivity and persuasiveness. This could be achieved through collaboration with the Nursing Assistant Association, which is a direct stakeholder in the nursing legislation, and other healthcare groups. Third, a more detailed analysis is required to understand the effects of media reports that focus on conflicts between interest groups and political confrontations, rather than on the public’s health needs and desires in the context of societal and demographic shifts. For instance, when minimum nurse-patient staffing legislation was proposed in the US state of California, numerous objections were raised based on concerns about increased medical costs and the inefficiency of nursing workforce management. Nevertheless, the law was enacted in 2004, prioritizing the value of improving patient safety and outcomes [33]. This enactment was supported by extensive research demonstrating the impact of the nursing workforce and the work environment on patient safety and outcomes [34]. In Korea, efforts should continue to provide more compelling evidence of how the nursing workforce, the nursing work environment, and nursing labor affect public health and safety. This evidence can be generated through collaborative research between nursing academia and clinical practice. Additionally, initiatives should be implemented to help the public gain a better understanding of the content of proposed legislation.

The limitations of this study are as follows. First, the data were sourced from 11 major newspapers in Korea, which does not encompass all media articles produced domestically and internationally. Consequently, it is challenging to assert that the findings reflect the overall direction of media coverage. Second, the topic modeling employed in this study relied on quantitative analysis methods, which precluded a qualitative and in-depth examination of the individual contents of the articles. Lastly, while newspaper articles provide objective data on social phenomena, constraints were present in subjectively judging the content or analyzing the emotional undertones. Henceforth, it is imperative to explore public sentiment, encompassing citizens’ reactions and emotions concerning nursing law, through the collection and analysis of social media data and interview data capturing citizens’ perspectives. Despite these limitations, this study is meaningful in that it presents objective evidence of the patterns and shifts in media coverage related to the proposed enactment of nursing legislation. This was achieved by analyzing relevant issues from the time the enactment of the nursing law emerged as a topic of interest until its ultimate rejection.

## Conclusion

This study revealed that during the study period, media coverage predominantly focused on the political and social disputes surrounding the passage of the proposed nursing legislation, rather than on the law’s necessity and specifics. Notably, the perspectives of the medical community—rather than those of the individuals directly impacted by the legislation, such as nurses and those receiving nursing care in the community—were prominently featured in the coverage. We posit that the campaign to pass the nursing law, which began to attract public attention due to the unique circumstances of the COVID-19 pandemic, did not maintain sufficient public interest because of the politicization of the issue and media emphasis on the perspectives of the medical community. Consequently, it is essential to assess whether the media has adequately conveyed the rationale behind the nursing legislation, its details, and the position of the nursing community. More compelling evidence is required to demonstrate the influence of the nursing workforce and the working conditions of nurses on public safety and health. Ultimately, a concerted effort should be made to enable the public to gain a better understanding of the provisions of this proposed nursing legislation.

## Supporting information

S1 Dataset The 991 articles underwent morpheme extraction. (XLSX)
